# Individual differences in the boldness of female zebrafish are associated with alterations in serotonin function

**DOI:** 10.1242/jeb.247483

**Published:** 2024-06-14

**Authors:** Fatemeh Beigloo, Cameron J. Davidson, Joseph Gjonaj, Shane A. Perrine, Justin W. Kenney

**Affiliations:** ^1^ Department of Biological Sciences, Wayne State University, Detroit, MI 48202, USA; ^2^ Department of Psychiatry and Behavioral Neurosciences, Wayne State University School of Medicine, Detroit, MI 48201, USA

**Keywords:** Risk taking, 5-HT, Neurotransmitter, SSRI, Monoamines, Personality

## Abstract

One of the most prevalent axes of behavioral variation in both humans and animals is risk taking, where individuals that are more willing to take risk are characterized as bold while those that are more reserved are regarded as shy. Brain monoamines (i.e. serotonin, dopamine and noradrenaline) have been found to play a role in a variety of behaviors related to risk taking. Using zebrafish, we investigated whether there was a relationship between monoamine function and boldness behavior during exploration of a novel tank. We found a correlation between serotonin metabolism (5-HIAA:5-HT ratio) and boldness during the initial exposure to the tank in female animals. The DOPAC:DA ratio correlated with boldness behavior on the third day in male fish. There was no relationship between boldness and noradrenaline. To probe differences in serotonergic function in bold and shy fish, we administered a selective serotonin reuptake inhibitor, escitalopram, and assessed exploratory behavior. We found that escitalopram had opposing effects on thigmotaxis in bold and shy female animals: the drug caused bold fish to spend more time near the center of the tank and shy fish spent more time near the periphery. Taken together, our findings indicate that variation in serotonergic function has sex-specific contributions to individual differences in risk-taking behavior.

## Teaser

**Summary:** Risk-taking behavior in female, but not male, zebrafish is regulated by the brain serotonergic system.

## INTRODUCTION

One of the most important and widely studied axes of behavioral variation is risk taking, where individuals that are more likely to take risks are characterized as bold, and those that are less likely to do so are described as shy (Budaev and Brown, 2011; Réale et al., 2007; Wilson et al., 1994). Such individual differences in behavior are prevalent throughout the animal kingdom and are thought to contribute to evolutionary fitness (Dingemanse and Wolf, 2013; Réale et al., 2007). For example, individual differences in risk taking have been found to affect dispersal in great tits (*Parus major*) (Dingemanse et al., 2003) and migration in roach fish (*Rutilus rutilus*) (Chapman et al., 2011). Furthermore, a recent meta-analysis found that higher risk taking in wild populations is associated with better survival (Moiron et al., 2020). In humans, increased risk taking in males is thought to contribute to their higher mortality compared with females (Kruger and Nesse, 2006; Wilson and Daly, 1985). However, despite our increased appreciation for the consequences of risk taking, we lack a detailed understanding of its biological basis.

Individual differences in the regulation of brain states by neuromodulators, such as monoamines, are potential contributors to behavioral variation. Monoamines [i.e. dopamine, noradrenaline (norepinephrine) and serotonin (5-hydroxytryptamine)] are a class of neurotransmitters that, despite being released from a small number of neuronal nuclei, have extensive projections throughout the brain and regulate the activity of many areas related to behavior (Jacobs and Azmitia, 1992). For example, serotonin has long been known to be involved in behaviors related to boldness, such as aggression and risk assessment (Homberg and Lesch, 2011; Lucki, 1998; Muller and Cunningham, 2020). Similarly, dopamine has also been implicated in risk taking due, in part, to its role in modulating reward salience (Schultz, 2010). Accordingly, specific genetic alleles that contribute to serotonergic and dopaminergic function are associated with individual differences in risk taking in humans and animals, albeit with some inconsistency (for review, see Montag and Reuter, 2014).

Understanding the biological basis for individual differences benefits from the use of model organisms where we have greater control over conditions and access to a variety of molecular, pharmacological and genetic tools. Zebrafish (*Danio rerio*) have recently gained popularity as a model organism for behavioral neurobiology because of their low cost, ease of genetic manipulation, domestication, availability, extensive behavioral repertoire and biological similarity to mammals (Gerlai, 2023; Kenney, 2020). Risk taking and boldness behavior in adult zebrafish has been characterized using a simple task called the novel tank test, where animals explore a new environment. Risky behavior in this context consists of exploring more of the tank and spending time in parts of the tank (e.g. the top) that would expose animals to greater risk of predation in the wild (Gerlai, 2020; Luca and Gerlai, 2012; Spence et al., 2006). Here, we tested the hypothesis that there is a relationship between boldness in a novel tank and monoamines in the brain of zebrafish.

## MATERIALS AND METHODS

### Subjects

Subjects were female and male zebrafish of the WIK strain that were within two generations of animals originally from the Zebrafish International Resource Center (ZIRC, catalog ID: ZL84) at the University of Oregon. Fish were 4–7 months of age and raised at Wayne State University. Housing was in high-density racks under standard conditions (water temperature: 27.5±0.5°C, salinity: 500±10 µS and pH 7.4±0.2) with a 14 h:10 h light:dark cycle (lights on at 08:00 h). Fish were fed twice daily, in the morning with a dry feed (Gemma 300, Skretting, Westbrook, ME, USA) and in the afternoon with brine shrimp (*Artemia salina*, Brine Shrimp Direct, Ogden, UT, USA).

The sex of fish was determined using three secondary sex characteristics: shape, color and presence of pectoral fin tubercles (McMillan et al., 2015). Sex was confirmed following euthanasia by the presence or absence of eggs. All procedures were approved by the Wayne State University Institutional Animal Care and Use Committee.

### Novel tank test

One week prior to behavioral manipulations, animals were placed into 2 l tanks with two female/male pairs per tank. Tanks were divided in half with a transparent divider, with one pair per side. Although there have been reports of dual housing arrangements resulting in increased aggression when combined with opaque tank dividers (Keck et al., 2015), we did not observe any evidence of excessive aggression (e.g. wounding or death). This arrangement allowed us to keep track of fish identity over days without social isolation or tagging. Our setup also ensured consistent housing conditions throughout experiments. This is important because housing conditions and sex ratios can have a large impact on behavior (Reolon et al., 2018; Soares et al., 2020). One hour before exposure to the novel tank, animals were removed from their housing racks and brought to the behavioral room to acclimate. Testing occurred between 11:00 h and 14:00 h. Following testing, fish remained in the behavioral room for at least 30 min before being returned to housing racks. Fish were individually placed into experimental tanks for 6 min while videos were recorded. Placement order was counterbalanced across sex and drug treatment (when applicable). Water was replaced between animals to prevent the buildup of chemical cues that may be released by fish during the test.

Novel tanks were five-sided (15×15×15 cm) and made from frosted acrylic (TAP Plastics, Stockton, CA, USA). Tanks were filled to a height of 12 cm with 2.5 l of fish facility water and placed in a white Plasticore enclosure to prevent disturbance by external stimuli and to diffuse light. The tanks were open on the top, above which D435 Intel RealSense^TM^ depth-sensing cameras (Intel, Santa Clara, CA, USA) were placed. Cameras were mounted 20 cm above the tanks and connected to Linux workstations using high-speed USB cables (NTC distributing, Santa Clara, CA, USA) for recording.

### Animal tracking and behavior

D435 cameras have color and depth video streams that create three-dimensional videos (Kuroda, 2018; Rajput et al., 2022). The color video was extracted from each file and five points along the fish were tracked using DeepLabCut (Mathis et al., 2018). Depth (i.e. distance from the camera) was calculated using the disparity between two infrared cameras. Three-dimensional swim traces were created by overlaying the tracking with the depth video using custom-written Python code. We extracted four behavioral parameters as previously described (Rajput et al., 2022): (1) distance from bottom, where a plane was fitted to the bottom of the tank and the distance from the fish to the plane was calculated; (2) center distance, where the distance of the fish to a line down the center of the tank was calculated; (3) distance traveled; and (4) percentage of tank explored, where the tank was divided into 1000 evenly spaced voxels and the proportion of voxels visited was calculated. Prior to calculations, swim traces were smoothed using a Savitzky–Golay filter with a length of seven frames and an order of three (Press and Teukolsky, 1990).

We also calculated a boldness index that combined bottom distance and percentage of tank explored. We did this by first calculating the *z*-scores for each variable within each sex and day of the novel tank test. Scores were then added to generate the boldness index. The combining of bottom distance and percentage of tank explored to capture boldness is based on theoretical and data-driven approaches. Theoretically, boldness is described as a willingness to take risk (Luchiari and Maximino, 2023; Toms et al., 2010; Wilson et al., 1994). In the novel tank test, risk taking is captured by a propensity to explore the new environment (i.e. percentage of tank explored) and a willingness to visit the top of the tank, which is considered more dangerous (i.e. bottom distance). A data-driven approach corroborated these theoretical concepts of boldness: unbiased clustering analysis of swim trajectories from over 400 fish of four different strains and both sexes found bold clusters characterized primarily by elevated percentage of tank explored and bottom distance (Rajput et al., 2022).

### HPLC

HPLC analysis was conducted as previously described (Davidson et al., 2022; Gheidi et al., 2023). Fish were removed from their housing racks and brought to the behavioral testing room at the same time of day as the behavioral experiments and allowed to acclimate for 1 h. Fish were then individually euthanized by placement in ice-cold water. Once movement ceased, fish were decapitated and the entire brain (excluding the spinal cord) was removed, flash frozen on dry ice and stored at −80°C. Whole brains were then thawed, weighed and homogenized by sonication (XL-2000, Misonix, Durham, NC, USA) for 3–5 s in 50 µl of 0.2 mol l^−1^ perchloric acid. Homogenate was visually inspected for uniform tissue suspension and then centrifuged at 10,000 ***g*** for 10 min at 4°C, and supernatant was carefully retained. A 20 µl aliquot of each sample was placed in a tube and fed into an autosampler of a Dionex Ultimate 3000 HPLC system at 5°C (Thermo Fisher Scientific, Waltham, MA, USA). External standards for dopamine (DA), serotonin (5-HT), noradrenaline, 3,4-dihydroxyphenylacetic acid (DOPAC) and 5-hydroxyindoleacetic acid (5-HIAA) were prepared via serial dilution (0.1 to 10 ng), including a blank of perchloric acid. Standards were processed in ascending concentrations and run in parallel and duplicate. Dopamine metabolites 3-methoxytyramine and homovanillic acid were not assessed because they could not be reliably detected.

Standards and samples were injected (10 µl) by an autoinjector using an acetonitrile-based MD-TM mobile phase (10% acetonitrile, Thermo Fisher Scientific). HPLC flow rate was 0.6 ml min^−1^. Samples passed through a reverse-phase column (Hypersil™ BDS C18 column, Thermo Fisher Scientifc) at 25°C, a guard cell (ESA guard cell, model 2050) set to 300 mV, and a column guard (2.1/3.0 mm i.d., Thermo Fisher Scientific). For electrochemical detection, we used an ultra-analytical dual-electrode cell (ESA, model 5011A) with a reference electrode set to −175 mV and a working electrode at 350 mV (gain of 100 µA for both). Detector values were captured using Chromileon 7 software (Dionex, Thermo Fisher Scientific) to quantify peak height, representing monoamine and metabolite levels. A detection threshold of 3 times the perchloric acid background was set. Samples that did not reach threshold were excluded from analysis. Standard curves for each chemical were generated to calculate levels and verify instrument stability (*R*^2^≥0.97). Levels of monoamines are expressed as ng mg^−1^ wet brain mass.

### Drug treatment

Escitalopram oxalate (Sigma-Aldrich, St Louis, MO, USA) was administered using a gelatin-based feed at a dose of 1 mg kg^−1^ body mass as previously described (Ochocki and Kenney, 2023). The gelatin feed consisted of 12% w/v gelatin (Sigma-Aldrich), 4% w/v spirulina (Argent Aquaculture, Redmond, WA, USA) and brine shrimp extract. The extract was made by suspending 250 mg ml^−1^ of mikro fine brine shrimp (Brine Shrimp Direct) in water followed by 1 h of stirring. The suspension was then centrifuged twice at 12,500***g***, keeping the supernatant each time, and then diluted in two volumes of water before addition to the gelatin feed mixture. Escitalopram stock (10 mg ml^−1^) or vehicle (water) was added prior to warming the solution to 45°C. After warming, drug- or vehicle-containing solution was pipetted into individually sized morsels for feeding at 1% body mass. Gelatin was allowed to set at −20°C for at least 20 min prior to feeding. We used this drug delivery method because it allows us to dose animals precisely based on their body mass (i.e. 1 mg kg^−1^) and requires less handling than other commonly used methods such as intraperitoneal injection or beaker dosing (Ochocki and Kenney, 2023).

Fish were given 2 days to acclimate to the gelatin feed prior to drug administration. On each day of the experiment, animals were removed from the housing racks to the behavioral room for 1 h before being given a non-dosed gelatin feed in lieu of their morning feed. Fish were briefly isolated by placement of transparent barriers in the tanks for 2–5 min. Once feed was given, we recorded whether fish ate the feed within 5 min. On day 1 of the novel tank test, all animals were given non-dosed feed 30 min prior to being placed in the novel tank. On day 2 of testing, fish were given either vehicle or escitalopram containing feed 30 min prior to being placed in the novel tank. Experimenters were unaware of the boldness category of the fish from day 1 because it was calculated after all data were collected. Only animals that ate the feed within 5 min were included in the analysis (2 female and 4 male fish given vehicle were excluded). Thirty minutes was chosen because several studies in zebrafish have found that oral drug administration results in peak serum concentrations within 30 min (Kim et al., 2017; Kulkarni et al., 2014; Zang et al., 2011) and prior work found large behavioral effects of drug administration using this method at this time point (Ochocki and Kenney, 2023).

### Statistical analysis

Data analysis was performed using version 4.3.0 of R (http://www.R-project.org/). Graphs were made using ggplot2 (Wickham, 2015). Statistical analysis was done using either a 2×3 (sex×day) mixed ANOVA, a 2×2 (drug×boldness) ANOVA, independent samples *t*-tests or Pearson's/Spearman's correlations as indicated. All tests were two-tailed. Interactions from ANOVA were followed up with pair-wise *t*-tests and corrected for multiple comparisons using the false discovery rate (FDR) (Benjamini and Hochberg, 1995). Sample sizes were determined to detect medium-sized effects with 80% power. Effect sizes for ANOVA are reported as η^2^ and *t*-tests as Cohen's *d*. The interpretation of effect sizes as small (0.01<η^2^<0.06; 0.2<*d*<0.5), medium (0.06≤η^2^<0.14; 0.5≤*d*<0.8) or large (η^2^≥0.14; *d*≥0.8) is based on Cohen (1988).

## RESULTS

### Sex differences in behavior and brain chemistry

**Fig. 1.**
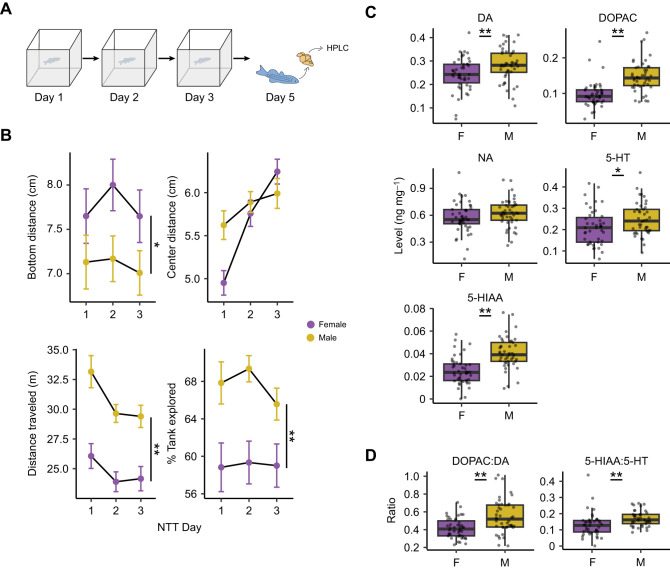
**Sex differences in behavior and brain chemistry.** (A) Experimental design for assessing the relationship between exploration of a novel tank and baseline brain chemistry. Fish were exposed to the novel tank on three consecutive days and brain tissue was collected for testing monoamine and metabolite levels using HPLC 2 days later. Image modified from Rajput et al. (2022). (B) The effect of sex and day on four exploratory behaviors in the novel tank. NTT, novel tank test. Asterisks indicate a main effect of sex from a sex×day ANOVA (*P<0.05, **P<0.001). Data are means±s.e.m. (C) The effect of sex on monoamine and metabolite levels. (D) The effect of sex on the ratio of monoamines to their metabolites. DA, dopamine; DOPAC, 3,4-dihydroxyphenylacetic acid; NA, noradrenaline; 5-HT, serotonin; 5-HIAA, 5-hydroxyindoleacetic acid. Boxplot center line is the median, hinges are interquartile ranges and whiskers are the hinge ±1.5 times the interquartile range. Asterisks indicate significance from independent sample *t*-tests (*P<0.05, **P<0.01). *n*=46 females, *n*=45 males.

To assess the relationship between baseline monoamine levels in the brain and exploratory behavior, we first exposed fish to a novel tank on three consecutive days and collected brain tissue 2 days after the last exposure (Fig. 1A). Three exposure days allowed us to determine whether the novelty of the environment, which is highest on day 1, contributes to any relationship between monoamines and boldness. Brains were dissected 2 days after the last behavioral test (day 5) to capture baseline monoamine and metabolite levels using HPLC. Initially, we determined whether there were sex differences in behavior and brain chemistry that would behoove us to analyze male and female fish separately (Fig. 1B–D). We measured four behaviors that captured different aspects of exploration during exposure to the novel tank: two predator avoidance behaviors (bottom dwelling as measured by bottom distance and thigmotaxis as measured by center distance: Blaser et al., 2010; Maximino et al., 2010; Spence et al., 2006), activity levels (distance traveled) and how much of the tank fish explored (Fig. 1B). Mixed two-way ANOVA (sex×day) were used to assess significance. For bottom distance, there was a small effect of sex (*P=*0.044, η^2^=0.029) where female fish spent more time near the top of the tank than males. There was no effect of day (*P=*0.44) or an interaction between sex and day (*P=*0.75). Center distance was not affected by sex (*P=*0.25), but there was a medium-sized effect of day (*P<*0.0001, η^2^=0.11) and a small interaction (*P=*0.034, η^2^=0.031). Both male and female fish increased their distance from the center over time, and on the first day, female fish spent more time closer to the center than male fish. Distance traveled also changed across days and differed between the sexes: there was a large effect of sex (*P<*0.001, η^2^=0.16), with male fish swimming further than female fish, and a small effect of day (*P<*0.001, η^2^=0.032), such that activity decreased over time. There was no sex by day interaction (*P=*0.51). Finally, there was a medium-sized effect of sex on percentage of tank explored (*P<*0.001, η^2^=0.082), where male fish explored more than female fish. There was neither an effect of day (*P=*0.50) nor a sex by day interaction (*P=*0.59) on percentage of tank explored.

We also determined whether there were sex differences in the brain levels of monoamines or their metabolites, 5-HIAA and DOPAC (Fig. 1C). We found a medium-sized effect of sex such that male fish had higher levels of dopamine (*P=*0.0082, *d*=0.57) and serotonin (*P=*0.032, *d*=0.47), and large effect on the metabolites DOPAC (*P<*0.001, *d*=1.23) and 5-HIAA (*P<*0.001, *d*=1.19). Levels of noradrenaline did not differ between the sexes (*P=*0.12). We also assessed the turnover of dopamine and serotonin by calculating the DOPAC:DA and 5-HIAA:5-HT ratios (Fig. 1D). We found that male fish had higher turnover than female fish for both neurotransmitters (DOPAC:DA: *P=*0.0001, *d*=0.87; 5-HIAA:5-HT: *P=*0.005, *d*=0.62). Because of these sex differences, male and female animals were analyzed separately throughout the study.

### Correlation between boldness and brain chemistry

**Fig. 2.**
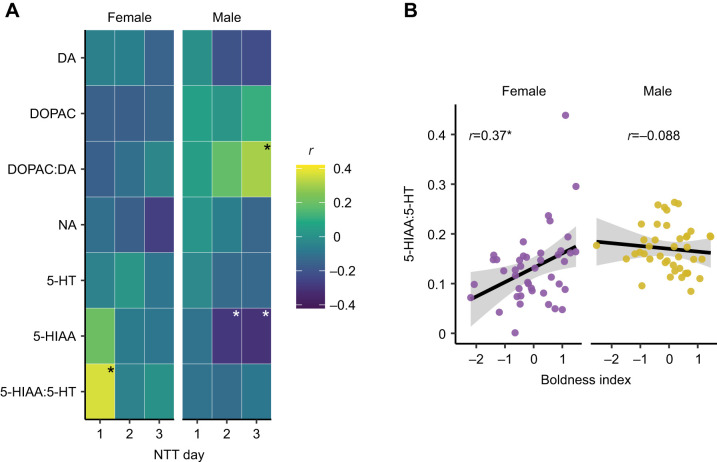
**Correlations between boldness index and brain chemistry.** (A) Pearson's correlations between the boldness index on each day of testing in the novel tank and monoamines, their metabolites, or the ratio of metabolites to monoamines. (B) Scatterplots and linear regressions for 5-HIAA:5-HT ratio and boldness index on day 1 of testing in the novel tank. *P<0.05.

To assess the relationship between brain monoamine levels and boldness, we calculated a boldness index for each animal that combined the *z*-scores of bottom distance and percentage of tank explored. Because of the sex differences we observed in behavior and brain chemistry (Fig. 1), the boldness index was calculated within sex so that we compared bold females with shy females and bold males with shy males. The boldness index is based on a previous study where we assessed behavior from over 400 animals and found that boldness in the novel tank test was composed of a combination of willingness to explore and proximity to the top of the tank (Rajput et al., 2022). This measure of boldness is consistent with its conceptual basis as a willingness to explore and take risks (Luchiari and Maximino, 2023; Réale et al., 2007; Toms et al., 2010; Wilson et al., 1994). We found boldness to be consistent across days of the task: Spearman's ρ calculations indicated that the rank of fish on the boldness index correlates across days in both females (day 1 to day 2: ρ=0.47, *P*=0.0012; day 2 to day 3: ρ=0.53, *P*=0.00019) and males (day 1 to day 2: ρ=0.33, *P*=0.027; day 2 to day 3: ρ=0.57, *P*<0.0001). We also determined whether monoamine and metabolite levels correlated with the boldness index on each day of testing (Fig. 2A). Behavior on the first day of the novel tank test was correlated with the 5-HIAA:5-HT ratio in female, but not male, zebrafish (female: *r*=0.37, *P=*0.016; male: *r*=−0.10, *P=*0.53). In female zebrafish, two animals had high levels of 5-HIAA:5-HT that might unduly influence the correlation (Fig. 2B), so we also calculated the non-parametric Spearman's ρ, which is less susceptible to outliers, with essentially the same result (ρ=0.36, *P=*0.02). There were also three significant correlations between boldness and brain chemistry in male fish, but none on the first day (Fig. 2A): on the second and third days, higher 5-HIAA levels were associated with less boldness (day 2: *r*=−0.29, *P=*0.050; day 3: *r*=−0.31, *P=*0.040). On the third day, the DOPAC:DA ratio correlated with boldness in male fish (*r*=0.30, *P=*0.043).

We also calculated correlations between individual behaviors and brain chemistry (Fig. S1). There were two other significant correlations in females, both for distance traveled (Fig. S1C): on day 1, we found a positive relationship with the 5-HIAA:5-HT ratio (*r*=0.32, *P=*0.036; Fig. S1C), and on day 2, we found a negative relationship with noradrenaline (*r*=−0.31, *P=*0.035). For male fish, there were no significant correlations on day 1 (Fig. S1). On days 2 and 3 there were several negative relationships with bottom distance (Fig. S1A): on day 2, with the 5-HIAA:5-HT ratio (*r*=−0.31, *P=*0.048), and with 5-HIAA on both day 2 (*r*=−0.45, *P=*0.0021) and day 3 (*r*=−0.40, *P=*0.0068). The only other significant correlations in males were with distance traveled on day 2 (Fig. S1C) where there was a positive relationship with the 5-HIAA:5-HT ratio (*r*=0.47, *P=*0.0015) and a negative relationship with 5-HT (*r*=−0.32, *P=*0.042).

### Blocking serotonin reuptake has opposing effects on thigmotaxis in bold and shy female zebrafish

**Fig. 3.**
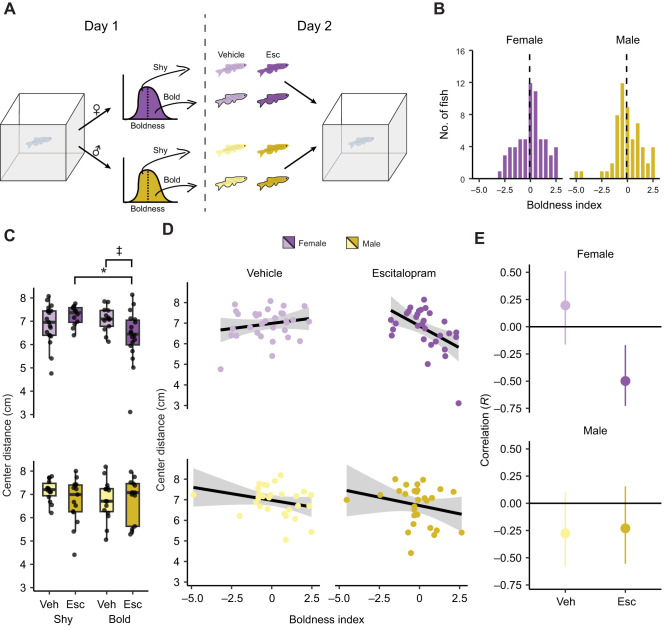
**The effect of escitalopram on the exploratory behavior of bold and shy fish.** (A) Fish were separated into bold and shy animals based on a median split of their boldness index during an initial exposure to the tank (day 1). On day 2, fish were given either vehicle (Veh) or 1 mg kg^−1^ escitalopram (Esc) prior to being placed back into the novel tank. (B) Histogram of boldness index on day 1 of exposure to the novel tank. The dashed line is the median. (C) Center distance on day 2 in female (top) and male (bottom) animals given either vehicle or escitalopram. Boxplot center is the median, hinges are interquartile ranges and whiskers are the hinge ±1.5 times the interquartile range. (D) Scatterplots and regression lines for boldness index on day 1 and center distance on day 2 for female and male fish given vehicle or escitalopram. (E) Pearson's correlations and 95% confidence intervals (error bars) corresponding to scatterplots in D. ^‡^*P<*0.10, *P<0.05 from false discovery rate (FDR)-corrected *post hoc t*-tests. Shy female: *n*=19 vehicle, *n*=12 escitalopram; bold female: *n*=13 vehicle, *n*=18 escitalopram; shy male: *n*=14 vehicle, *n*=15 escitalopram; bold male: *n*=15 vehicle, *n*=13 escitalopram.

The correlation between boldness and the 5-HIAA:5-HT ratio in female fish on day 1 suggests a role for the serotonergic system in risk-taking behavior. We focused on the findings from day 1 because the tank is most novel on this day and novelty is considered an important element for the expression of boldness (Gosling, 2001; Réale et al., 2007; Toms et al., 2010; Wilson et al., 1994). Based on these findings, we hypothesized that bold and shy female fish, as assessed during an initial exposure to the tank, would respond differently to a drug that targets the serotonergic system. To test this hypothesis, we administered escitalopram, a selective serotonin reuptake inhibitor (SSRI) (Sánchez et al., 2004), prior to exploration of the tank (Fig. 3A). To identify bold and shy fish, we did a median split on the boldness index within sex from behavior during an initial exposure to the novel tank (Fig. 3B). A day later, fish were administered either vehicle or 1 mg kg^−1^ escitalopram, 30 min before being placed back into the tank. We assessed four behaviors on the second day (bottom distance, center distance, distance traveled and percentage of tank explored) using a 2×2 (drug×boldness) ANOVA design.

The effect of escitalopram on center distance in female fish depended on whether animals were categorized as bold or shy (Fig. 3C, top), as evidenced by a medium-sized interaction between boldness and drug (*P=*0.014, η^2^=0.10): shy animals given escitalopram increased their center distance whereas bold fish decreased theirs. Follow-up FDR-corrected pair-wise *t*-tests found a difference between bold and shy animals given escitalopram (*P=*0.038) and a trend towards a difference between bold fish given vehicle and escitalopram (*P=*0.061). There were no main effects of boldness (*P=*0.11) or escitalopram (*P=*0.41). In male fish, there were no effects of the drug (*P=*0.33), boldness (*P=*0.47) or an interaction between drug and boldness (*P=*0.32; Fig. 3C, bottom).

We also determined whether boldness on day 1 correlated with center distance on day 2 and whether this was affected by escitalopram treatment (Fig. 3D,E). In vehicle-treated female fish, there was no correlation between boldness on day 1 and center distance on day 2 (*r*=0.19, *P=*0.28) whereas there was a moderate negative correlation in those given escitalopram (*r*=−0.50, *P=*0.005). The 95% confidence intervals for vehicle- or escitalopram-treated female fish slightly overlapped (Fig. 3E, top). In male fish, there were no correlations between boldness index on day 1 and center distance on day 2 in animals given vehicle (*r*=−0.28, *P=*0.15) or escitalopram (*r*=−0.23, *P=*0.23). The 95% confidence intervals for vehicle- and escitalopram-treated males largely overlapped (Fig. 3E, bottom).

There were no interactions between boldness and drug for the other behavioral parameters (Fig. S2). For bottom distance (Fig. S2A), we found a medium-sized effect of boldness in female fish (*P=*0.033, η^2^=0.067) where bolder fish spent more time near the top of the tank. There was no effect of drug treatment (*P=*0.71) or an interaction with boldness (*P=*0.22). In male fish, bottom distance was not affected by day 1 boldness (*P=*0.15) or drug (*P=*0.17) and there was no interaction (*P=*0.19). Locomotor activity (Fig. S2B) was not affected by escitalopram in female fish (*P=*0.15). In male fish, there was a medium-sized effect of drug (*P=*0.02, η^2^=0.096), where fish given escitalopram swam less. There were no effects of boldness or an interaction between boldness and drug on locomotor activity in either female or male fish (female: boldness: *P=*0.83, interaction, *P=*0.31. male: boldness: *P=*0.35, interaction: *P=*0.96). The percentage of the tank explored on day 2 (Fig. S2C) was not affected by boldness on day 1 in female fish (*P=*0.45) or drug treatment (*P=*0.11) and there was no interaction between boldness and drug treatment (*P=*0.35). In male fish, there was a trend towards a small effect of boldness on percentage of tank explored (*P=*0.061, η^2^=0.55) such that bold fish explored more than shy fish (Fig. 3F, bottom). There was also a trend towards a small effect of escitalopram (*P=*0.08, η^2^=0.53) but no interaction between boldness and drug (*P=*0.86).

We also determined whether escitalopram affected correlations between bottom distance, distance traveled and percentage of tank explored on day 2 and boldness index on day 1 (Fig. S3). In vehicle-treated female fish, there was a moderate positive correlation between day 1 boldness index and day 2 bottom distance (*r*=0.58, *P<*0.001; Fig. S3A, top). For bottom distance, the 95% confidence intervals between vehicle- and escitalopram-treated female fish overlapped slightly (Fig. S3A, top). There was no correlation between boldness index on day 1 and percentage of tank explored, or distance traveled in female fish (Fig. S3B, top). In male fish, there were no significant correlations between boldness index on day 1 and behavior on day 2, and all 95% confidence intervals in vehicle- and escitalopram-treated fish had large overlaps (Fig. S3, bottom).

## DISCUSSION

Our findings identify the serotonergic system as playing a sex-specific role in individual differences in the boldness behavior of zebrafish. This conclusion is based on several of our results: (1) we found a correlation between boldness during an initial exposure to a novel tank and serotonin turnover in the brain that was limited to female fish (Fig. 2). (2) Altering serotonin at the synapse of female fish using an SSRI, escitalopram, resulted in decreased thigmotaxis in bold animals and increased thigmotaxis in shy animals (Fig. 3C); there was no such interaction in males. (3) Escitalopram treatment affected correlations between boldness during an initial exposure to a novel tank and behavior on a second exposure in female, but not male, zebrafish (Fig. 3D,E; Fig. S3). Taken together, our data suggests an important role for serotonin in the emergence of individual differences in risk taking during exploration of a novel environment, particularly in female animals.

The serotonergic system of zebrafish is characterized by several populations of neurons, some of which resemble those seen in mammals, such as the raphe nuclei, and others that appear to be unique to fish, such as clusters in the hypothalamus and pretectum (reviewed in Lillesaar, 2011). Despite these differences in neuroanatomy, the behavioral functions of the serotonergic system are remarkably conserved, with serotonin being implicated in aggression, fear and anxiety in both zebrafish and mammals (Herculano and Maximino, 2014; Lillesaar, 2011; Lucki, 1998; Muller and Cunningham, 2020; Tea et al., 2024; Winberg and Thörnqvist, 2016). This may be due to the fact that, as in mammals, many long-range serotonergic projections in zebrafish originate from the raphe nuclei (Lillesaar et al., 2009) and some of these projections in mammals, which are missing in zebrafish, contain local populations of serotonergic neurons that may subserve a similar function (Lillesaar, 2011). Given this high level of conservation, findings from the present study are likely to provide insight into serotonergic function across species.

The most striking finding from this study is that bold and shy female zebrafish respond in opposite ways to treatment with escitalopram: drug-treated bold fish spent more time in the center of the tank whereas the drug caused shy fish spent more time in the periphery (Fig. 3). This echoes work in mammals, where individual differences in the open field activity of rats were associated with opposing effects of different serotonergic drugs on time to enter the open arms of an elevated T-maze (Verheij et al., 2009), and in humans, where the acute effects of modifying serotonergic function on moral judgment and social emotions depended on personality traits (Crockett et al., 2010; Kanen et al., 2021). However, the question remains: how does altering serotonin at the synapse result in differential behavioral responses in bold and shy animals? One likely explanation is that differences in serotonergic receptor expression and/or localization throughout the brain contribute to bold and shy animals responding differently to escitalopram. Serotonin receptors are highly conserved and are divided into four classes based on their signaling mechanisms (Nichols and Nichols, 2008). Most receptors are G-protein coupled, except for 5-HT_3_, which is a cation-permeable ligand-gated ion channel. Further complicating the picture in zebrafish is that, because of the fish-specific genome duplication event that occurred ∼350 million years ago (Meyer and Van de Peer, 2005), many serotonin receptors have multiple isoforms unique to fish (Maximino et al., 2016; Norton et al., 2008). Nonetheless, it is known that modulation of different classes of serotonergic receptors has opposite influences on the exploratory behavior of zebrafish: 5-HT_1A_ and 5-HT_1B/D_ antagonists increase bottom distance whereas a 5-HT_2_ receptor antagonist decreases this behavior (Maximino et al., 2013; Nowicki et al., 2014). Thus, it is likely that variation in the expression of 5-HT receptors throughout the brain underlies individual differences in boldness.

We also found that the ratio of 5-HIAA:5-HT correlated with boldness in female zebrafish such that animals with a higher ratio were bolder than those with a lower ratio. This higher turnover could reflect differences in the synthesis or degradation of serotonin. Degradation of 5-HT to 5-HIAA is catalyzed by monoamine oxidase, for which zebrafish have only one gene instead of two as in mammals (Setini et al., 2005). In contrast, zebrafish have four genes that encode the rate-limiting enzyme for serotonin synthesis, tryptophan hydroxylase (*tph1a*, *tph1b*, *tph2* and *tph3*), compared with two in mammals (Ren et al., 2013; Walther and Bader, 2003). It is likely that these three isoforms have different kinetics, although only the kinetics of *tph3* (formerly known as *th2*) are currently known (Ren et al., 2013). The expression of these isoforms varies throughout the brain (Bellipanni et al., 2002; Lillesaar et al., 2007; Ren et al., 2013; Teraoka et al., 2004), and thus it may be the case that the differences in the turnover of 5-HT in bold and shy fish reflects distinct distributions of tryptophan hydroxylase isoforms.

Two prior studies have examined links between behavioral variation and monoamine levels in the brains of zebrafish. Tran and colleagues (2016) found that zebrafish with high locomotor activity had lower levels of brain serotonin, with no difference in dopamine. We also observed a negative correlation between distance traveled and serotonin, albeit only in male fish and only on the second day of exposure to the tank (Fig. S1C). Another study found a correlation between serotonin and time spent in the top of a novel tank (Maximino et al., 2013), an effect we did not observe (Fig. S1A). Several methodological differences may explain the discrepancies between the present study and prior work. In the present study, brain dissections occurred 2 days after the last behavioral manipulation to capture baseline levels of chemicals in the brain to uncover the presence of constitutive differences in monoamines. In contrast, both prior studies collected samples immediately after exposure to a novel tank. Given that exposure to the novel tank test itself is known to elicit region-specific elevations in serotonin (Maximino et al., 2013), a direct comparison of our findings with those of these previous studies is difficult. It may be the case that serotonin levels increased from baseline in some animals more than others in response to the behavioral manipulation. In addition, we found several sex differences in behavior and monoamine levels, with, for example, the relationship between locomotor activity and serotonin being restricted to male fish. The prior studies did not indicate sex ratios or stratify results by sex (Maximino et al., 2013; Tran et al., 2016). Nonetheless, taken together, these findings support the conclusion that serotonin contributes to individual differences in the exploratory behavior of zebrafish.

The link between serotonin levels in the brain and variation in boldness-related behaviors is not limited to zebrafish, but has also been found in other fish species, rodents, and invertebrates such as crayfish. For example, similar to our findings, bold European sea bass (*Dicentrarchus labrax*) and hatchery-reared Atlantic cod (*Gadus morhua*) have higher 5-HIAA:5-HT ratios than their shy counterparts (Alfonso et al., 2019; Meager et al., 2012). In male rats (*Rattus norvegicus*), animals that spend less time in the open arms of an elevated plus maze, and thus are considered more anxious or shy, have higher levels of serotonin in the amygdala, but not in other brain regions such as the hippocampus or striatum (Näslund et al., 2015). This brain region specificity in rats may be why we did not observe a similar relationship in male fish in our study, as we used whole-brain homogenates. In crayfish, levels of serotonin, but not dopamine, were higher in animals that spend more time in darker parts of a maze than less anxiety-provoking light parts (Fossat et al., 2015). These findings across phyla speak to the high evolutionary conservation of the contribution of serotonin to individual behavioral variation despite wide variation in neuroanatomy (Backström and Winberg, 2017; Herculano and Maximino, 2014).

Prior work using escitalopram, or another SSRI, fluoxetine, found that acute administration via water exposure alters behavior in the novel tank test, increasing time spent near the top of the tank (Fontana et al., 2022; Lima-Maximino et al., 2020; Maximino et al., 2013; Sackerman et al., 2010). Here, however, we did not find that escitalopram increased bottom distance (Fig. S2A). This is likely because we administered escitalopram prior to a second exposure to the tank, when the tank was no longer novel (Fig. 3A). This suggests that novelty may be an important element that engages the serotonergic system. A link between novelty and serotonin is further supported by our finding that the correlation between boldness and serotonin turnover was only present during the initial exposure to the novel tank, but not on subsequent days (Fig. 2A). Interestingly, polymorphisms in serotonin transporter genes in humans are associated with novelty seeking, often in a sex-specific manner (Kazantseva et al., 2008; Vormfelde et al., 2006). Thus, it may be that novelty is important for engaging the serotonergic system in the context of risk taking and its importance may differ between sexes.

We found several differences between male and female fish: the link between serotonin and boldness was limited to female zebrafish, and females had lower levels of most neurotransmitters and metabolites (except for noradrenaline), and lower DOPAC:DA and 5-HIAA:5-HT ratios than males. Similarly, Dahlbom and colleagues (2012) found that females had lower 5-HIAA:5-HT and DOPAC:DA ratios in the forebrain, and lower serotonin in the hindbrain, but elevated dopamine in the forebrain. The difference in dopamine levels between prior work and the present study may be due to our use of whole-brain homogenates compared with dividing the brain into the forebrain and hindbrain. Indeed, regional heterogeneity in sex differences in monoamine levels has been reported in rodents (Bowman et al., 2009; De la Fuente et al., 2003; Liiver et al., 2023), suggesting that it is a widespread phenomenon. These sex differences may reflect differences in tryptophan and/or serotonin metabolism due to the regulation of related genes by sex hormones (Barth et al., 2015). How these sex differences in neurochemistry give rise to sexual dimorphism in behavior will likely require a closer examination of regional differences.

We also found a potential role for dopamine in boldness as the DOPAC:DA ratio was correlated with boldness in male fish, but only on day 3 of exposure to the novel tank (Fig. 2A). Because the correlation in males appeared to strengthen during each exposure to the tank, this suggests that its contribution to boldness may increase as the environment becomes more familiar, and less novel. Prior work has also implicated dopamine in boldness and related behaviors in zebrafish. For example, Thörnqvist and colleagues (2019) reported that bold zebrafish had higher expression levels of some dopamine receptors (*drd2a* and *drd2b*), and Kacprzak et al. (2017) found that genetic removal of the dopamine transporter results in elevated bottom dwelling. They also found that administration of a broad-spectrum dopamine antagonist reduced time spent at the bottom of the tank (Kacprzak et al., 2017). Interestingly, both of these studies used male animals and assessed boldness-related behavior during extended exposure times: Thörnqvist et al. (2019) used a 15 min exposure to a novel tank (compared with our 3×6 min exposures per day) and Kacprzak et al. (2017) documented behavior over two full days in their genetic studies and 2 h in their pharmacological manipulations. Taken together with the present study, these findings suggest that the contribution of dopamine to boldness may be most prominent in males and when an environment is more familiar.

The present study has several limitations that should be acknowledged. For example, we used whole-brain homogenates for HPLC to identify monoamines and their metabolites. Future work should explore potential regional differences for the contribution of the serotonergic system to zebrafish behavior in more detail by taking advantage of recent advances in tools for whole-brain mapping in adult zebrafish (Kenney et al., 2021). Another limitation to the present work is the use of escitalopram, an SSRI, which is expected to alter serotonin levels at synapses throughout the brain. We used this broad approach expecting that it was most likely to reveal differences that may be attributable to multiple aspects of serotonergic function. A fruitful avenue for future work might be to use receptor-specific drugs in bold and shy animals or to knock out receptor genes using recent advances in CRISPR/Cas9 (Kroll et al., 2021). Another limitation is that the present study used only one measure of boldness; a more complete picture of boldness would be captured by considering other tests such as willingness to explore a novel object (Dahlbom et al., 2011). Finally, the use of a domesticated model organism may limit the generalizability of our findings to animals in the wild. This is because the domestication process results in reduced genetic variation and associated changes in behavior and morphology (Whiteley et al., 2011; Wright et al., 2006; Zala et al., 2012).

The suggested involvement of serotonin in personality and individual behavioral differences goes back nearly half a century (Murphy et al., 1977; Schooler et al., 1978; Zuckerman et al., 1984). However, a mechanistic understanding of how serotonin contributes to behavioral variation has remained elusive. By uncovering a role for serotonin in the individual differences in boldness of zebrafish, the present study lays the foundation for using the rapidly growing toolbox available to zebrafish researchers to shed light on how variation in serotonergic function gives rise to individual differences in behavior.

## Supplementary Material

10.1242/jexbio.247483_sup1 Supplementary information
